# Prevalence of sarcopenia under different diagnostic criteria and the changes in muscle mass, muscle strength, and physical function with age in Chinese old adults

**DOI:** 10.1186/s12877-022-03601-7

**Published:** 2022-11-22

**Authors:** Mengyu Cao, Junsong Lian, Xisheng Lin, Jinwei Liu, Chao Chen, Shuaixuan Xu, Shuang Ma, Fang Wang, Nihui Zhang, Xiaolei Qi, Guogang Xu, Nan Peng

**Affiliations:** 1 Department of Rehabilitation Medicine, The Second Medical Center & National Clinical Research Center for Geriatric Diseases, Chinese PLA General Hospital, Beijing, China; 2 The Ninth Health Care Department, The Second Medical Center & National Clinical Research Center for Geriatric Diseases, Chinese PLA General Hospital, Beijing, China; 3 Medical school of Chinese PLA, Chinese PLA General Hospital, Beijing, China; 4 Institute of Psychiatry and Neuroscience Xinxiang Medical University, Xinxiang, Henan China; 5 Nursing Department, The Sixth Medical Center, Chinese PLA General Hospital, Beijing, China; 6 The Second Medical Center & National Clinical Research Center for Geriatric Diseases, Chinese PLA General Hospital, Beijing, China

**Keywords:** Sarcopenia, Muscle mass, Muscle strength, Physical function, Diagnosis

## Abstract

**Background:**

At present, there are several diagnostic criteria of sarcopenia were used in China, and the diagnostic criteria were not unified. This study aims to investigate the consistency between the latest sarcopenia diagnostic criteria Asian Working Group for Sarcopenia(AWGS 2019) and other common diagnostic criteria. The changes of muscle mass, muscle strength and physical function with age and their effects on the diagnosis of sarcopenia were also analyzed.

**Methods:**

A total of 1009 men aged ≥60 years were enrolled from multiple communities. Skeletal muscle mass index, grip strength and 6 m gait speed were measured. The consistency of AWGS 2019 with other diagnostic criteria was analyzed and the trends of these three indicators were observed. The differences of muscle mass, muscle strength and function among different diagnostic criteria and age groups were evaluated. In addition, the change trends of these three indicators with age were observed.

**Results:**

According to AWGS 2019 diagnostic criteria, the incidence of sarcopenia in male aged 60–69 years, 70–79 years and over 80 years was 1.5%, 9.6% and 33.1%, respectively. AWGS 2019 was highly consistent with other diagnostic criteria (*Kappa* = 0.66–0.80, *P* < 0.01), except the Foundation for the National Institutes of Health(FNIH) (*Kappa* = 0.32, *P* < 0.01). When AWGSA2019 diagnostic criteria are applied, the prevalence of decreased muscle strength (39.1%) and physical function (46.4%) was significantly higher than that of low muscle mass (35.9%) in the men over 80 years old. Muscle strength (*P* < 0.01) and function (*P* < 0.01) decreased at the same rate with age, both of which were more significant than muscle mass (*P* < 0.01).

**Conclusion:**

AWGS 2019 was highly consistent with other criteria. Maintaining muscle mass should be the focus of attention before age 80, while improving muscle strength and function should be focused after age 80 to prevent disability.

## Background

Aging of population is a serious challenge in China. The seventh national census show that the number of people aged ≥60 accounts for 18.7% of the total population, and the number of disabled people reaches 52.71 million [1]. According to a demographic study by Peking University [2], it is estimated that by 2030, there will be more than 77 million disabled old adults in China with a disability period of 7.44 years, and by 2050, the proportion of disabled people will increase to more than 70% if without prevention and control measures. Sarcopenia is generally defined as age-associated loss of skeletal muscle mass accompanied by decreased muscle strength and/or decreased body function, is one of the major concerns in the old adults in recent years. Sarcopenia significantly increases the risk of fall, prolonged bed rest, developing chronic disease, re-hospitalization, physical disability and even death in the elder population [3, 4]. Therefore, early diagnosis and prevention of sarcopenia have attracted much attention.

The diagnostic criteria of sarcopenia are not uniform, and the diagnostic procedures of several sarcopenia working groups are constantly being updated. In recent years, the European and Asian sarcopenia working groups have updated the diagnostic criteria, but there is a lack of large-scale epidemiological studies in China that apply the latest diagnostic criteria. In addition, the changes of muscle mass, muscle strength and physical function, which are important diagnostic indicators of sarcopenia closely related to aging, are still barely known.

Therefore, this study focused on the old adults in the community and compared the efficacy of the latest diagnostic criteria of sarcopenia. The changes of muscle mass, muscle strength and function with age were analyzed.

## Methods

### Study participants

This study initially screened 1446 old adults from multiple communities in different districts (including Haidian District, Chaoyang District, Fengtai District, Dongcheng District and Xicheng District) of Beijing, China from April to July 2019. The primary community health centers affiliated with the PLA General Hospital invited people aged 60 years from these communities to participate in the study. Because the characteristics of the population lead to the low number of women recruited, and previous research have found that the prevalence of sarcopenia varies greatly by sex. Therefore, to avoid gender differences in the overall prevalence of sarcopenia, we excluded female and included only male in the study.

Inclusion criteria were: 1) Male subjects aged ≥60 years, 2) Subjects had the ability to complete various test projects, 3) Subjects who voluntarily participated in and cooperated with the research. Exclusion criteria were: 1) Subjects with severe bone, joint or muscle diseases with pain and limited activity, 2) Subjects who could not stand by themselves, 3) Subjects with heart, lung or other important organ diseases in acute or terminal stage, 4) Subjects with severe cognitive or communication disorders, or surgical history within 5 or 6 months, 6) Subjects with pacemaker implantation or other metal implantation,7) Subjects being resident in 24-hour care institutions.

According to the inclusion and exclusion criteria, a total of 437 people were excluded. Female subjects (76), subjects under 60 years old (253), and subjects who did not meet the inclusion criteria (108) were excluded. Therefore, a total of 1009 males were finally included in this study. Their age ranged from 60 to 98 years old, with an average age of 73.9 ± 8.65 years old and a median age of 73 years old. The study complied to the criterion of the Declaration of Helsinki. The research proposal was authorized by the Ethics Committee and the Institutional Review Committee of Hospital (Approval Number: S2019–140-01). Each patient was told about the study and signed the Informed Consent.

### Measurements of sarcopenia

#### Muscle mass

Skeletal muscle mass was determined with body composition analyzer (Model Inbody770, Seoul Bodi Co., LTD., South Korea). Subjects should fast as much as possible and metal objects such as necklaces and watches should be removed. Bioelectrical impedance analysis was used to measure appendicular skeletal muscle mass (ASM) in extremities [5]. The skeletal muscle mass index (SMI) was calculated with formula: SMI = ASM/ height^2^.

#### Muscle strength

The handgrip strength test was performed using a hand-held electronic grip meter (JAMAR, Sammons Preston). The subjects were instructed to press hard for 3 to 5 seconds until the number stops rising [6]. The handgrip strength test was conducted for 3 times and the maximum value of the dominant hand was recorded. Subjects rested for 1 ~ 2 min after each measurement.

#### Physical function

For the gait speed test, 10 m measuring space was reserved and the subjects were instructed to walk the whole distance at their daily pace. Timing started at 2 m and ended at 8 m [7]. The walking time of 6 m was measured, and the gait speed was calculated. Walking was permitted while using a walking aid such as a stick or frame.

### Diagnostic criteria

In this study, 6 different diagnostic criteria that are currently most widely used were selected to diagnose sarcopenia, including the Asian Working Group for Sarcopenia (AWGS2014 [8] and AWGS2019 [9]), European Working Group on Sarcopenia in Older People (EWGSOP1 [10] and EWGSOP2 [11]), the International Working Group on Sarcopenia [12] (IWGS) and the Foundation for the National Institutes of Health [13] (FNIH). The details of these diagnostic criteria are shown in Table 1.

**Table 1:** Different diagnostic criteria of sarcopenia^a^

	①Muscle mass (SMI)	②Muscle strength (Handgrip strength)	③Physical function (Gait speed)	Diagnosis
AWGS2019	< 7.0 kg/m^2^	< 28 kg	< 1 m/s	① + ②/① + ③
EWGSOP2	< 7.0 kg/m^2^	< 27 kg	≤0.8 m/s	① + ②
AWGS 2014	< 7.0 kg/m^2^	< 26 kg	≤0.8 m/s	① + ②/① + ③
EWGSOP1	< 7.23 kg/m^2^	< 30 kg	≤0.8 m/s	① + ②/① + ③
IWGS	< 7.23 kg/m^2^	**–**	< 1 m/s	① + ③
FNIH	ALM_BMI_ < 0.789	< 26 kg	≤0.8 m/s	① + ② + ③

*SMI* skeletal muscle mass index, *AWGS* Asian Working Group for Sarcopenia, *EWGSOP* European Working Group on Sarcopenia in Older People, *IWGS* the International Working Group on Sarcopenia, *FNIH* the Foundation for the National Institutes of Health

^a^The cut-off values in the above diagnostic criteria were all males. The cut-off values for females were not listed separately

### Statistical methods

Statistical analysis was conducted using SPSS 17.0(IBM, Chicago, IL). The consistency of AWGS2019 with other diagnostic criteria was analyzed by Kappa value, positive predictive value (PPA), and negative predictive value (NPA). Group analysis of low muscle mass (LMM), low muscle strength (LMS) and low physical function (LPF) was performed according to different diagnostic criteria and ages, and the differences among groups were detected by Chi-square test. Linear regression analysis was used to assess and compare age-related changes in muscle mass, muscle strength, and physical function. In order to compare the reduction of the age-related indicators in different age groups, all variables were divided by the standard value (cut-off value) and standardized [14]. A *P*-value < 0.05 was considered statistically significant.

## Results

### Clinical characteristics of participants

The enrolled participants were divided into 60–69 years old group (*n* = 396), 70–79 years old group (*n* = 365) and over 80 years old group (*n* = 248). The statistical results of various measurement indexes (age, height, weight, SMI, handgrip strength and gait speed) in all age groups are shown in Table 2. The average SMI, handgrip strength and gait speed of the total old adults were respectively (7.59 ± 0.69) kg/m^2^, (36.47 ± 8.01) kg and (1.26 ± 0.27) m/s. With the increase of age, SMI, handgrip strength and gait speed of the old adults showed varying degrees of decline.

**Table 2:** Baseline characteristics of the subjects (*N* = 1009)

	60–69 years (***n*** = 396)	70–79 years (***n*** = 365)	Over 80 years (***n*** = 248)	Total (***N*** = 1009)
**Age (year)**	65.5 ± 2.49	74.7 ± 2.70	86.0 ± 4.69	73.9 ± 8.65
**Height (cm)**	172.60 ± 5.19	170.84 ± 4.97	168.46 ± 5.45	170.9 ± 5.42
**Weight (kg)**	74.70 ± 9.60	72.45 ± 9.28	69.07 ± 9.90	72.5 ± 9.80
**SMI (kg/m**^**2**^**)**	7.82 ± 0.59	7.60 ± 0.64	7.20 ± 0.73	7.59 ± 0.69
**Handgrip strength (kg)**	40.87 ± 6.65	36.43 ± 6.60	29.51 ± 6.88	36.47 ± 8.01
**Gait speed(m/s)**	1.41 ± 0.20	1.27 ± 0.21	1.01 ± 0.28	1.26 ± 0.27
**FTCST(s)**	9.29 ± 2.67	10.70 ± 3.13	13.94 ± 4.56	10.94 ± 3.83
**Balance test(s)**				
**side by side stand**	10.00 ± 0.00	10.00 ± 0.00	9.8 ± 1.17	9.9 ± 0.95
**semi-tandem stand**	9.94 ± 0.43	9.90 ± 0.57	9.5 ± 1.68	9.81 ± 0.95
**tandem stand**	9.48 ± 2.82	9.53 ± 1.55	8.32 ± 2.65	9.21 ± 2.44
**SPPB (score)**	11.53 ± 0.83	11.23 ± 1.07	9.68 ± 12.61	10.97 ± 1.53

*SMI* skeletal muscle mass index, *FTCST* five-time chair stand test, *SPPB* Short Physical Performance Battery

### The incidence of sarcopenia under different diagnostic criteria

**Fig. 1:**
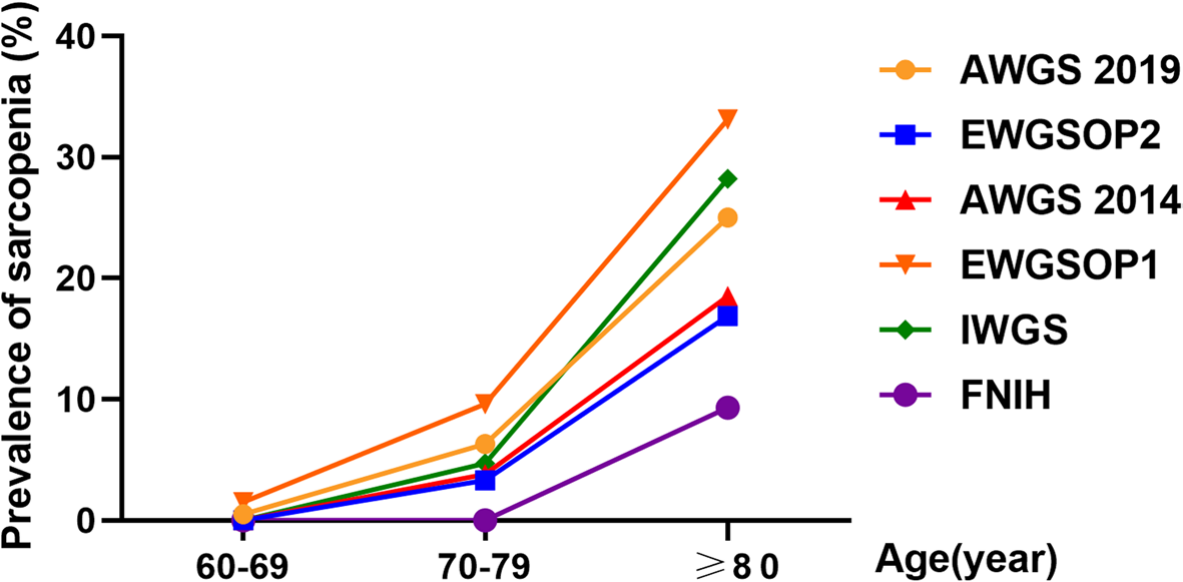
Comparison of the prevalence of sarcopenia in different age groups under different diagnostic criteria. *AWGS*: Asian Working Group for Sarcopenia; *EWGSOP*: European Working Group on Sarcopenia in Older People; *IWGS*: the International Working Group on Sarcopenia; *FNIH*: the Foundation for the National Institutes of Health

The old adults were diagnosed with sarcopenia based on different criteria, and stratified statistics were carried out according to age. According to the diagnostic criteria of AWGS2019, AWGS 2014, EWGSOP2, EWGSOP1, IWGS and FNIH, the number of cases diagnosed with sarcopenia were 87 cases (8.6%), 60 cases (5.9%), 54 cases (5.4%), 123 cases (12.2%), 87 cases (8.6%) and 23 cases (2.3%), respectively. The highest number was observed under EWGSOP1 criteria and the lowest number was found under FNIH criteria. The prevalence of sarcopenia increased with age, especially among those over 80 years old (Fig. 1). The prevalence of sarcopenia was 0–1.5% in 60–69 years old group, 0–9.6% in 70–79 years old group and 9.3–33.1% in over 80 years old group. In addition, there was a remarkable increase in the incidence of sarcopenia diagnosed by IWGS criteria, second only to EWGSOP1 criteria, in those over 80.

### Consistency analysis between AWGS 2019 and other diagnostic criteria

The AWGS2019 criteria had a wide range of PPA compared with the other five diagnostic criteria, ranging from 21.8% for FNIH to 83.9% for EWGSOP1 (Table 3). The NPA was high in all criteria, with the NPA reaching 100% in EWGSOP2 and AWGS 2014. AWGS2019 was highly consistent with the other four criteria except FNIH criteria (*Kappa* = 0.661–0.802, *P* < 0.001).

**Table 3:** Analysis of consistency between AWGS 2019 and other diagnostic criteria

	***PPA*** (%)	***NPA*** (%)	***Kappa*** value	***P*** value
**EWGSOP2**	62.1	100	0.749	< 0.001^*^
**AWGS 2014**	69.0	100	0.802	< 0.001^*^
**EWGSOP1**	83.9	94.6	0.661	< 0.001^*^
**IWGS**	70.1	97.2	0.673	< 0.001^*^
**FNIH**	21.8	99.6	0.321	< 0.001^*^

*PPA* positive predictive value, *NPA* negative predictive value, *AWGS* Asian Working Group for Sarcopenia, *EWGSOP* European Working Group on Sarcopenia in Older People, *IWGS* the International Working Group on Sarcopenia, *FNIH* the Foundation for the National Institutes of Health

^*^P < 0.001

### Prevalence of LMM, LMS and LPF

The cut-off values of LMM, LMS and LPF were slightly different under different diagnostic criteria (Table 1). The SMI cut-off value under EWGSOP1 and FNIH criteria was 0.23 kg/m^2^ higher than that of other criteria, and the prevalence of LMM was increased from 17 to 29% (Table 4). EWGSOP2 reduced the cut-off value of grip strength from 30 kg to 27 kg, reduced the prevalence of LMS by 7.6%, and reduced the prevalence of sarcopenia by 6.8%. Meanwhile, after AWGS2019 update, the critical value of grip strength was increased by 2 kg, and the cut-off value of gait speed was also increased by 0.2 m/s. The results showed that the incidence of LMS, LPF and sarcopenia increased by 4.5%, 8.5%, and 2.7%, respectively (Table 4).

**Table 4:** Prevalence of LMM, LMS and LPF under different diagnostic criteria

	LMM	LMS	LPF	Sarcopenia
**AWGS 2019**	172 (17.0%)	145 (14.4%)	160 (15.9%)	87 (8.6%)
**EWGSOP 2**	172 (17.0%)	121 (12%)	75 (7.4%)	54 (5.4%)
**AWGS 2014**	172 (17.0%)	100 (9.9%)	75 (7.4%)	60 (5.9%)
**EWGSOP 1**	293 (29.0%)	198 (19.6%)	75 (7.4%)	123 (12.2%)
**IWGS**	293 (29.0%)	–	160 (15.9%)	87 (8.6%)
**FNIH**	198 (19.6%)	100 (9.9%)	75 (7.4%)	23 (2.3%)
***χ2***	105.72	58.02	103.86	88.15
***P *value**	< 0.001^*^	< 0.001^*^	< 0.001^*^	< 0.001^*^

*LMM* low muscle mass, *LMS* low muscle strength, *LPF* low physical function, *AWGS* Asian Working Group for Sarcopenia, *EWGSOP* European Working Group on Sarcopenia in Older People, *IWGS* the International Working Group on Sarcopenia, *FNIH* the Foundation for the National Institutes of Health

^*^P < 0.05

### Age-related changes of muscle mass, muscle strength and physical function

**Fig. 2:**
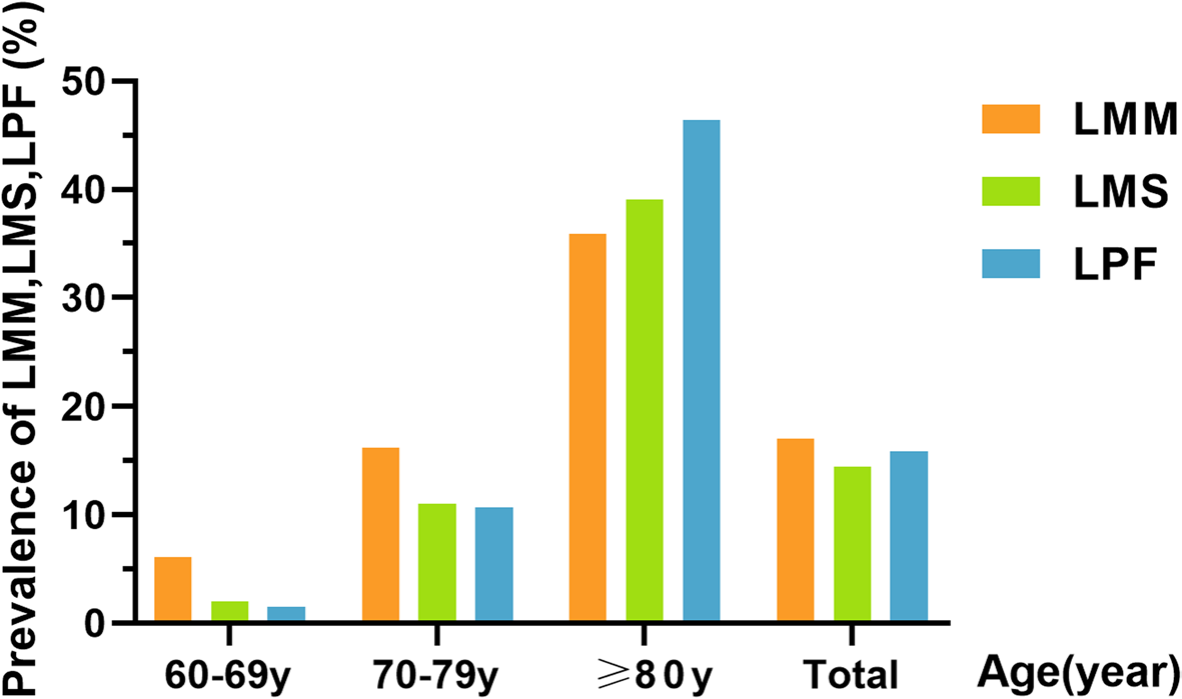
Prevalence of LMM, LMS, and LPF in different age groups. Muscle mass, muscle strength and physical function were assessed by SMI, grip strength, and gait speed, respectively. *LMM*: low muscle mass; *LMS*: low muscle strength; *LPF*: low physical function

**Fig. 3:**
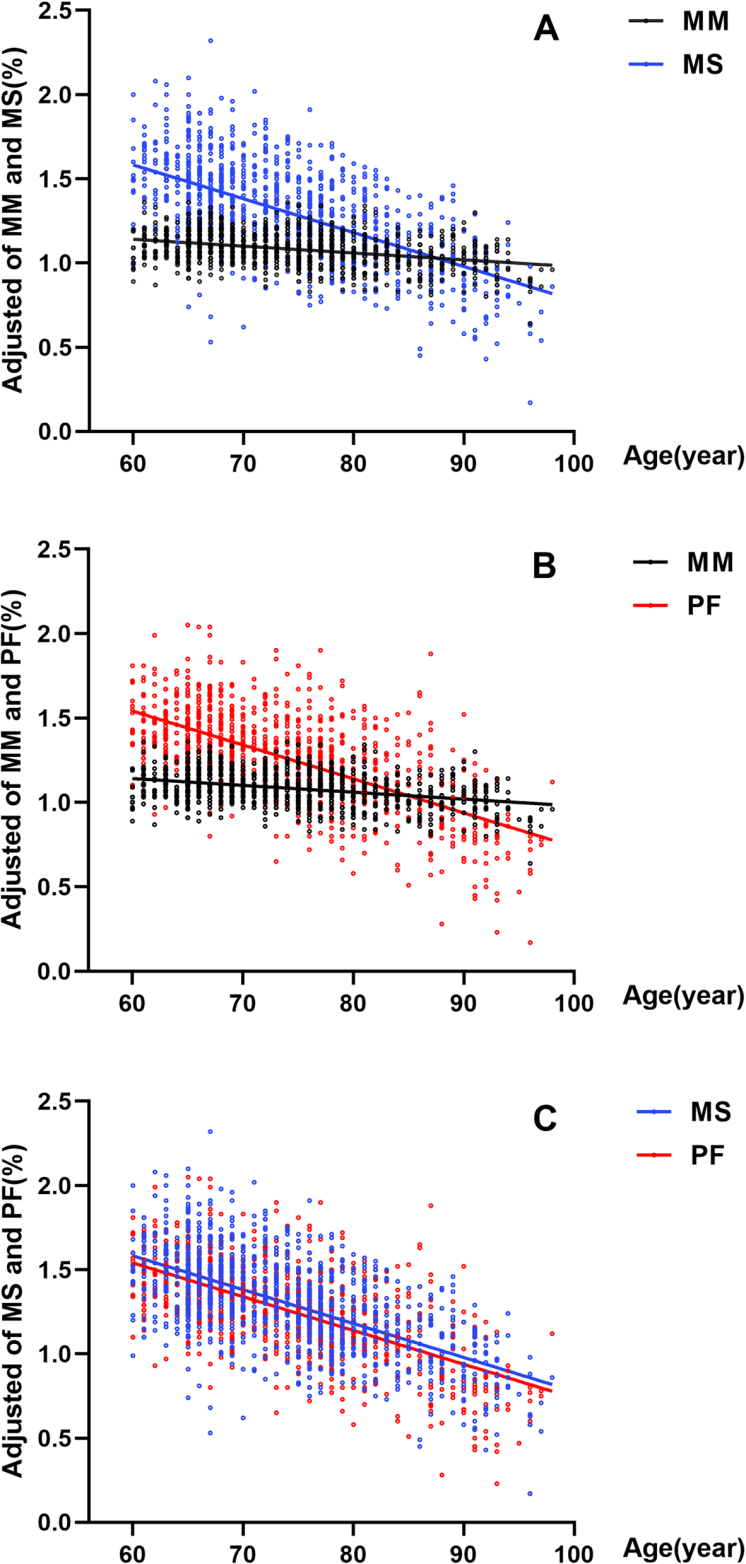
Changes of muscle mass, muscle strength and physical function with age. **A** and **B** show that muscle mass (assessed by SMI) is not in parallel with muscle strength (assessed by grip strength) and function (assessed by gait speed), and the decline of muscle strength and function is more significant than the decline of muscle mass. **C** shows that muscle strength is in parallel with the decline of function. *MM*: muscle mass; *MS*: muscle strength; *PF*: physical function

LMM, LMS and LPF were defined by AWGS2019 criteria. With the increase of age, the prevalence of LMM, LMS and LPF gradually increased (Fig. 2). The total prevalence of LMM was 17%, which was higher than that of LMS (14.4%) and that of the LPF (15.9%). The prevalence of LMM, LMS and LPF in the group over 80 years old were respectively 35.9, 39.1 and 46.4%. Muscle strength (*β* = − 0.004, *P* < 0.001) and physical function (*β* = − 0.02, *P* < 0.001) decreased faster with age than muscle mass (*β* = − 0.004, *P* < 0.001) (Fig. 3).

## Discussion

At present, a number of international sarcopenia working groups have issued expert consensus and diagnostic criteria. The diagnostic criteria are also constantly being updated and improved. We compared the latest criteria of the AWGS2019 with other diagnostic criteria in this study, and found that the incidence of sarcopenia under different criteria varied from 2.3 to 12.2%, with the highest prevalence diagnosed by EWGSOP1 criteria and the lowest prevalence diagnosed by FNIH. Our results is accordance with the prevalence of sarcopenia reported by current studies (8–50%) [15]. One of the main reasons may be that the diagnostic criteria for sarcopenia are inconsistent. A recent study in South Korea investigated 2313 community old adults people aged 70–84, and found that the prevalence of sarcopenia was 1.5–21% [16], slightly higher than this study. The average age of the subjects was also higher than that in our study. Another study in China compared AWGS2019 with other diagnostic criteria and found significant difference in prevalence, ranging from 18.1% in FNIH criteria to 57.1% in EWGSOP1 criteria [17]. The higher prevalence may be due to the fact that most of the study subjects lived in poor villages in Western China and the medical resources were inadequate. They found that the prevalence of sarcopenia diagnosed by EWGSOP2 was 6.5%, significantly lower than EWGSOP1, AWGS 2014, and IWGS (22.3%, 10.9%, 24.5%), but higher than FNIH (6.0%) [18], which was consistent with the results of this study.

We found that AWGS 2019 was highly consistent with other criteria except FNIH for the diagnosis of sarcopenia, with *Kappa values* as high as 0.802 and 0.749 in AWGS 2014 and EWGSOP 2, respectively. The diagnostic consistency between AWGS 2019 and FNIH was the worst, possibly because the diagnostic criteria of FNIH for sarcopenia were more stringent, requiring simultaneous reduction of muscle mass, grip strength and physical function, while the cut-off values of the two diagnostic criteria were significantly different. Previous studies compared EWGSOP 2 criteria with AWGS, EWGSOP 1, IWGS and FNIH, respectively, in the old adults in the Chinese community, which showed that EWGSOP 2 was not consistent with other criteria in the diagnosis of sarcopenia (*Kappa value* = 0.159–0.592). The consistency with IWGS criteria is the worst [18]. This study showed that AWGS 2019 had good diagnostic consistency in our study population. However, because the AWGS 2019 criteria were newly revised, further studies are needed for verification and improvement.

The revision of diagnostic criteria mainly lies in the adjustment of cut-off value, and the main reason for the difference in prevalence is the change of cut-off value. Therefore, the analysis of the influence of cut-off value change on the prevalence of sarcopenia can provide a scientific basis for the diagnosis of sarcopenia suitable for the old adults in China in the future. In the new AWGS 2019, the original threshold for SMI (< 7.0 kg/cm^2^) was retained, but the cut-off value for male grip strength was increased from < 26 kg to < 28 kg. There has been evidence that the prevalence of LMS in male sarcopenia patients was higher (22.1%) when the cut-off value of grip strength < 28 kg, compared with when the cut-off value < 26 kg (12.9%) [16]. In this study, when the cut-off value increased from 26 kg to 27 kg, 28 kg and 30 kg, the prevalence of LMS increased by 2.1%, 2.4% and 5.2%, respectively. Therefore, these differences in male prevalence may be related to different diagnostic cut-off values for grip strength. In addition, in AWGS 2019, the cut-off value of LPF increased from≤0.8 m/s to < 1 m/s, and the prevalence of LPF can be increased from 5.7 to 23.6% [16], which is similar to the results of this study. If the cut-off value of gait speed is increased by 0.2 m/s, the prevalence could be increased by 8.5%. Therefore, slight adjustment of the cut-off value can significantly affect the diagnosis of sarcopenia. Due to regional, ethnic and national differences, the cut-off values developed by various working groups may be not applicable for Chinese elder individuals. Therefore, it is particularly important to identity the cut-off values suitable for the diagnosis of sarcopenia in China, which is also the focus of our future research.

With age, muscle strength and function declined faster than muscle mass. Under 80 years of age, muscle mass decreased more, while muscle strength and function began to decline rapidly over 80 years of age. Skeletal muscle is used to maintain energy consumption when other sources of energy are depleted, so reduced muscle mass can significantly increase morbidity and mortality from otherwise survivable diseases [19]. The old adults with LPF and LMS are more vulnerable to become disability and frailty. When they face mild stress (such as replacement of drugs, minimally invasive surgery, etc.), they are more likely to stay in bed, which may induce acute onset of chronic diseases, or increase the hospitalization rate and mortality, thus leading to further deterioration of muscle strength and function, and resulting in a vicious cycle [20–22]. Therefore, early targeted intervention measures should be given to the old adults with different ages and physical functions to prevent the occurrence and development of sarcopenia.

Another result of this study indirectly confirmed that functional decline was more significant in the old adults. Compared with EWGSOP1, which had the highest prevalence of sarcopenia, IWGS increased the functional cut-off value (gait speed < 1 m/s vs ≤0.8 m/s) and maintained the same muscle mass cut-off value (SMI < 7.23 kg/m^2^), but the diagnostic prevalence was still lower in the old adults under 80 years old, even lower than AWGS2019 criteria (SMI < 7.0 kg/m^2^). This may be because muscle mass loss is more common in old adults under 80 years old, while the prevalence of sarcopenia is relatively low even with increased diagnostic cut-off values for low physical function. However, when the gait speed of the old adults decreased significantly after 80 years old, the prevalence of IWGS began to increase significantly, which was almost consistent with the prevalence of EWGSOP1.

## Conclusions

In this study, it was found that the decline of muscle strength and function was more obvious than that of muscle mass in the elder male population. Before the age of 80, the change of muscle mass should be closely monitored to avoid the reduction of muscle mass. After the age of 80, more attention should be paid to functional improvement to prevent disability. It suggests that we should provide targeted preventive intervention measures for different ages and functional states of the old adults in clinical practice.

## Data Availability

The datasets generated and/or analyzed during the current study are not the publicly available due presence of private information but are available from the corresponding author on reasonable request.

## Abbreviations

ASM: Appendicular Skeletal Muscle Mass
SMI: Skeletal Muscle Mass Index
AWGS: Asian Working Group for Sarcopenia
EWGSOP: European Working Group on Sarcopenia in Older People
IWGS: The International Working Group on Sarcopenia
FNIH: The Foundation for the National Institutes of Health
PPA: Positive Predictive Value
NPA: Negative Predictive Value
LMM: Low Muscle Mass
LMS: Low Muscle Strength
LPF: Low Physical Function
MM: Muscle Mass
MS: Muscle Strength
PF: Physical Function

## References

[1] National Bureau of Statistics. Main data of the seventh national population census [EB/OL]. (2021-05-11) [2021-08-15]. http://www.stats.gov.cn/ztjc/zdtjgz/zgrkpc/dqcrkpc/ggl/202105/t20210519_1817693.html.

[2] Luo Y, Su B, Zheng X (2021). Trends and challenges for population and health during population aging - China, 2015-2050. China CDC Wkly.

[3] Cruz-Jentoft AJ, Sayer AA (2019). Sarcopenia. Lancet.

[4] Beaudart C, McCloskey E, Bruyère O, Cesari M, Rolland Y, Rizzoli R (2016). Sarcopenia in daily practice: assessment and management. BMC Geriatr.

[5] Jeon KC, Kim SY, Jiang FL, Chung S, Ambegaonkar JP, Park JH (2020). Prediction equations of the multifrequency standing and supine bioimpedance for appendicular skeletal muscle mass in Korean older people. Int J Environ Res Public Health.

[6] Roberts HC, Denison HJ, Martin HJ, Patel HP, Syddall H, Cooper C (2011). A review of the measurement of grip strength in clinical and epidemiological studies: towards a standardised approach. Age Ageing.

[7] Guralnik JM, Ferrucci L, Pieper CF, Leveille SG, Markides KS, Ostir GV (2000). Lower extremity function and subsequent disability: consistency across studies, predictive models, and value of gait speed alone compared with the short physical performance battery. J Gerontol A Biol Sci Med Sci.

[8] Chen LK, Liu LK, Woo J, Assantachai P, Auyeung TW, Bahyah KS (2014). Sarcopenia in Asia: consensus report of the Asian working Group for Sarcopenia. J Am Med Dir Assoc.

[9] Chen LK, Woo J, Assantachai P, Auyeung TW, Chou MY, Iijima K (2020). Asian working Group for Sarcopenia: 2019 consensus update on sarcopenia diagnosis and treatment. J Am Med Dir Assoc.

[10] Cruz-Jentoft AJ, Baeyens JP, Bauer JM, Boirie Y, Cederholm T, Landi F (2010). European working group on sarcopenia in older people. Sarcopenia: European consensus on definition and diagnosis: report of the European working group on sarcopenia in older people. Age Ageing.

[11] Cruz-Jentoft AJ, Bahat G, Bauer J, Boirie Y, Bruyère O, Cederholm T (2019). Writing Group for the European Working Group on sarcopenia in older people 2 (EWGSOP2), and the extended group for EWGSOP2. Sarcopenia: revised European consensus on definition and diagnosis. Age Ageing.

[12] Fielding RA, Vellas B, Evans WJ, Bhasin S, Morley JE, Newman AB (2011). Sarcopenia: an undiagnosed condition in older adults. Current consensus definition: prevalence, etiology, and consequences. International working group on sarcopenia. J Am Med Dir Assoc.

[13] Studenski SA, Peters KW, Alley DE, Cawthon PM, McLean RR, Harris TB (2014). The FNIH sarcopenia project: rationale, study description, conference recommendations, and final estimates. J Gerontol A Biol Sci Med Sci.

[14] Bai HJ, Sun JQ, Chen M, Xu DF, Xie H, Yu ZW (2016). Age-related decline in skeletal muscle mass and function among elderly men and women in Shanghai, China: a cross sectional study. Asia Pac J Clin Nutr.

[15] Arango-Lopera VE, Arroyo P, Gutiérrez-Robledo LM, Pérez-Zepeda MU, Cesari M (2013). Mortality as an adverse outcome of sarcopenia. J Nutr Health Aging.

[16] Kim M, Won CW (2020). Sarcopenia in Korean community-dwelling adults aged 70 years and older: application of screening and diagnostic tools from the Asian working Group for Sarcopenia 2019 update. J Am Med Dir Assoc.

[17] Liu X, Hou L, Zhao W, Xia X, Hu F, Zhang G (2021). The comparison of sarcopenia diagnostic criteria using AWGS 2019 with the other five criteria in West China. Gerontology..

[18] Yang L, Yao X, Shen J, Sun G, Sun Q, Tian X (2020). Comparison of revised EWGSOP criteria and four other diagnostic criteria of sarcopenia in Chinese community-dwelling elderly residents. Exp Gerontol.

[19] Nishigori T, Tsunoda S, Obama K, Hisamori S, Hashimoto K, Itatani Y (2018). Optimal cutoff values of skeletal muscle index to define sarcopenia for prediction of survival in patients with advanced gastric Cancer. Ann Surg Oncol.

[20] Walston J, Buta B, Xue QL (2018). Frailty screening and interventions: considerations for clinical practice. Clin Geriatr Med.

[21] Dent E, Martin FC, Bergman H, Woo J, Romero-Ortuno R, Walston JD (2019). Management of frailty: opportunities, challenges, and future directions. Lancet..

[22] Nascimento CM, Ingles M, Salvador-Pascual A, Cominetti MR, Gomez-Cabrera MC, Viña J (2019). Sarcopenia, frailty and their prevention by exercise. Free Radic Biol Med.

